# Ultrasonographic manifestations and the effective diagnosis of epididymal leiomyosarcoma: Case report and systematic literature review

**DOI:** 10.3389/fonc.2023.1101801

**Published:** 2023-02-10

**Authors:** Ruixiao Song, Jing Xi, Honglei Shi, Zhixin Xue, Huifang Li, Xiaolong Yu

**Affiliations:** ^1^ Department of Ultrasonics, Wujin Hospital Affiliated with Jiangsu University, Changzhou, Jiangsu, China; ^2^ Science and Education Section, The Wujin Clinical College of Xuzhou Medical University, Changzhou, Jiangsu, China; ^3^ Department of Urology, Wujin Hospital Affiliated with Jiangsu University, Changzhou, Jiangsu, China; ^4^ Department of Pathology, Wujin Hospital Affiliated with Jiangsu University, Changzhou, Jiangsu, China

**Keywords:** leiomyosarcoma, epididymis, ultrasonographic, diagnosis, antidiastole

## Abstract

**Background:**

Epididymal leiomyosarcoma is an extremely rare tumor. In this study, we describe the sonographic features of this uncommon tumor.

**Methods:**

A case of epididymal leiomyosarcoma diagnosed at our institute was retrospectively analyzed. Ultrasonic images, noted clinical manifestations, treatment procedures, and pathology results were collected for this patient. The same information was collected from a systematic literature search on epididymal leiomyosarcoma, including the PUBMED, Web of Science and Google Scholar databases.

**Results:**

The literature search resulted in 12 articles; we were able to extract data from 13 cases of epididymal leiomyosarcomatosis. The median patient age was 66 (35–78) years, and the average tumor diameter was 2–7 cm. All patients had unilateral epididymal involvement. The lesions were all solid, irregular-shaped in almost half of the cases, featured clear borders in six cases, and had unclear borders in four cases. The internal echogenicity was heterogeneous in the majority of lesions: six cases were hypoechoic (7/11) and three cases moderately echoic (3/10). Information on blood flow within the mass was provided in four cases, but all were noted with significant vascularity. Surrounding tissue invasion was discussed in 11 cases, with 4 featuring peripheral invasion or metastasis.

**Conclusion:**

Epididymal Leiomyosarcoma demonstrates sonographic characteristics common to many malignant tumors, such as increased density, irregular shape, heterogeneous internal echogenicity, and hypervascular. Ultrasonography is helpful to differentiate benign epididymal lesions, and can provide some reference for clinical diagnosis and treatment. However, compared with other malignant tumors of the epididymis, it has no characteristic sonographic features,and pathological confirmation is required.

## 1 Introduction

Sarcoma accounts for only 1%–2% of primary malignant tumors of the male genitourinary system (1). Leiomyosarcoma is a malignant mesenchymal tumor of smooth muscle. Paratesticular leiomyosarcoma mostly originate from the tunica testis and spermatic cord. Only 2% of these tumors arise from the epididymis (2), making epididymal leiomyosarcoma (EL) the rarest malignant urogenital tumor. Clinical examination findings of EL are similar to those of benign tumors. It is difficult to differentiate EL from other scrotal tumors based on physical examination and clinical symptoms alone, and the misdiagnosis rate is high. EL is prone to early hematogenous diffusion (3), and prognosis varies greatly according to the degree of differentiation. To date, no standard treatment scheme has been established. Rodriguez D et al.reported that tumor grade, stage, histological type, lymph node metastasis, and distant metastasis were independent prognostic indicators of paratesticular leiomyosarcoma (4). Kolev et al.proposed that EL recurrence is common; therefore, early diagnosis and differential diagnosis are very important (5).

Ultrasonography provides high resolution and real-time, dynamic images; it is the first choice for diagnosis of scrotal diseases. In the past, ultrasound findings of epididymal leiomyosarcoma were limited to case reports, and the description was relatively simple. In this study, we collected ultrasound imaging findings from patients with confirmed EL: one patient from our own institution, and the remaining patients *via* an extensive literature search. Reviewed the ultrasound images of a patient with EL diagnosed at our institution, and summarized the ultrasound manifestations of epididymal leiomyosarcoma in the existing literature. We aimed to determine whether there are ultrasound imaging features can be used to assist clinical diagnosis, and provide a reference for treatment.

## 2 Methods

We retrospectively collected data from a patient at our institution with epididymal leiomyosarcoma, including basic patient information, clinical manifestations, ultrasonic imaging manifestations, treatment procedures, and pathological results.

**Figure 1:**
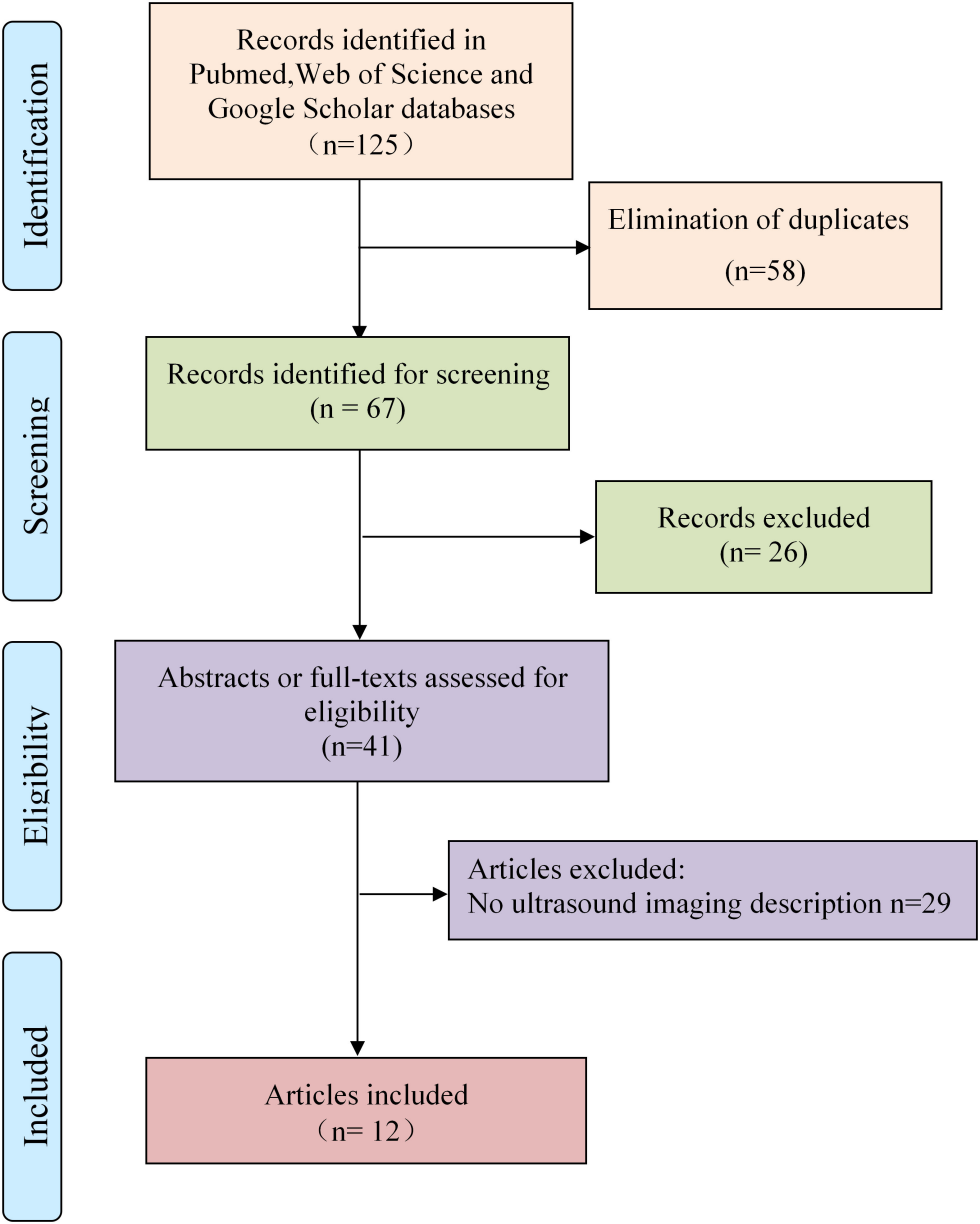
Study flow diagram.

In July 2022, we systematically reviewed the literature on epididymal leiomyosarcoma in PUBMED, the Web of Science and Google scholar. The search terms used were “Leiomyosarcoma” and “Epididymis,” with the “full text” filter utilized to ensure that no relevant papers were excluded. We used the system evaluation and meta-analysis (PRISMA) preferred reporting project flowchart (**Figure 1**). After eliminating duplicates, two independent authors (Xiaolong Yu and Ruixiao Song) evaluated the abstracts and full texts of the selected publications using the following qualification criteria: 1) articles on epididymal leiomyosarcoma published in English, and 2) discussion of clinical and ultrasonic imaging manifestations, treatment procedures, and disease pathology. Any differences in study selection were overcome through discussion.

### 2.1 Statistical analysis

Qualitative data is presented as numbers and percentages. Quantitative data is presented as mean with standard deviation (SD), or median and interquartile range (IQR).

## 3 Results

### 3.1 Case report

**Figure 2:**
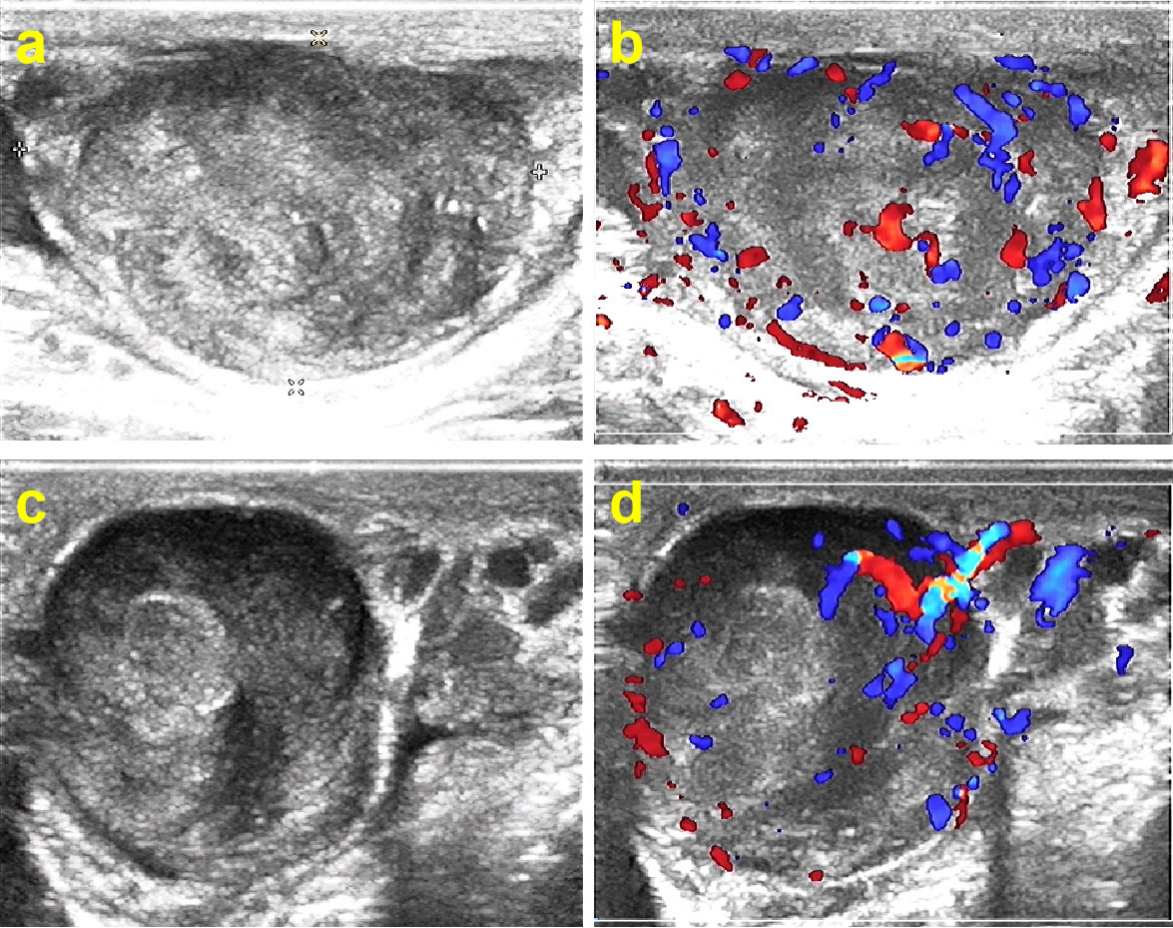
Ultrasonography of leiomyosarcoma in the caudal epididymis, **(A, B)** longitudinal scan, **(C, D)** transverse scan. There is a solid hypoechoic mass with heterogeneous internal echogenicity, clear boundaries, and abundant blood flow signals.

A 58-year-old man presented with a painless mass in the left epididymis without obvious inducement 1 year ago. Recently, the mass had enlarged, and he was admitted to our hospital on June, 2022. On examination, there were no renal masses, distention, or flank/ureteral pathway tenderness on palpation. There was no suprapubic tenderness or percussive dullness. External genitalia was visually normal, and the right testis and epididymis were normal; however, a 3 × 4 cm hard knot was palpated in the left epididymis. Blood results were normal, and tumor markers were negative. On ultrasound imaging, an heterogeneous hypoechoic mass was seen in the tail of the left epididymis, approximately 3.0 × 2.0 cm in size, with a clear boundary, unsmooth margin, local angulation, and no obvious lateral shadow, Doppler US revealed that the tumors were hypervascular. (**Figure 2**). No obvious abnormalities were found in the thorax, abdomen, and pelvis *via* computerized tomography (CT).

The patient underwent resection of the mass in the left epididymis at Wujin Hospital Affiliated with Jiangsu University. During the surgery, a solid tumor was found at the tail of the left epididymis, with a maximum diameter of approximately 3 cm and an unclear boundary. The tumor was gray, braided, tough, and unclear. Pathology demonstrated a smooth muscle tumor, suggestive of a highly differentiated leiomyosarcoma. Microscopic hematoxylin and eosin staining showed high numbers of cells with spindle, whirlpool, and braided arrangements. There was obvious cell heterogeneity with visible mitotic figures: eosinophilic cytoplasm, rod-shaped nuclei with some irregularities and visible nucleoli, rough nuclear chromatin, and an increased nucleocytoplasmic ratio.

**Figure 3:**
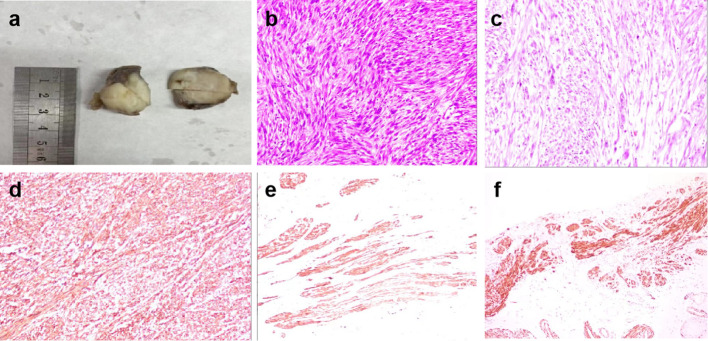
**(A)** The gross specimen of the tumor is grayish white, braided, tough and poorly defined. **(B)** Hematoxylin and eosin-stained slices show spindle-shaped or pleomorphic tumor cells, with obvious cell atypia and more mitotic figures. **(C)** Eosinophilic cytoplasm of the tumor cells. The nuclei are rod-shaped, round, or cigar-like at both ends. The chromatin is rough and the nucleoplasmic ratio is increased, with some irregular nuclei. Nucleoli and mitotic figures are visible, and interstitial inflammatory cells are scattered and infiltrated. **(D)** Caldesomon is diffusely and positively expressed in the tumor cells. **(E)** Desmin is diffusely and strongly expressed in the tumor cells. **(F)** SMA is positively expressed in the epididymal smooth muscle cells.

Immunohistochemistry showed that caldesomon and desmin were strongly expressed in the tumor cells. In the epididymal tissue, expression of smooth muscle actin (SMA), prostate-specific antigen (PSA), and compound prostate-specific antigen (CPSA) had increased (**Figure 3**). The expression of CD43, HMB 45, CD117 (C-kit), and CD34 was negative.

One month after the operation, the patient was re-admitted for radical resection of the left testis and epididymis. Postoperative pathology showed no residual tumor cells in the left testis, epididymis, and spermatic cord. No chemotherapy or radiotherapy was administered to the patient. Periodic axial computed tomography scans of the thorax, abdomen, and pelvis have remained normal, and he has remained free of recurrence for the past 6 months.

### 3.2 General patient information

In July 2022, we systematically reviewed the literature on epididymal leiomyosarcoma, searching the PUBMED and Web of Science databases. We screened11 studies featuring 12 cases, and also included the case from our institution, resulting in total 13 cases. The median age of the patients was 66 (35–78) years, and the average diameter of the tumor was 2–7 cm. All patients had unilateral epididymal involvement, including five cases with the right epididymis affected, six with the left epididymis affected, and two with unspecified locations. Twelve cases described the invasion information of the surrounding tissues, of which five cases featured peripheral invasion and seven cases featured local tumor growth only. Intraoperative information was supplied for two invasive tumors and one localized tumor.

### 3.3 Ultrasonic imaging characteristics

Thirteen cases of epididymal leiomyosarcoma examined using ultrasound were solitary masses, with all lesions appearing solid (100%). Among the 11 cases with echoic description, most masses (eleven cases) were hypoechoic (63,6%), three were isoechoic 27.3%), and one was hyperechoic (9.1%) when compared to the testis. Seven cases described echoic homogeneity, including one case with homogeneous echogenicity (14.3%) and six cases with heterogeneous echogenicity (85.7%). The shape of the tumor was described in ten cases: four round or oval (40%) and six irregular (60%) tumors. Ten cases included a description of the mass boundary, of which six tumors had clear boundaries (60%) and four had unclear boundaries (40%). Only four cases described the blood flow inside the mass, but all of them were hypervascular (100%) (**Table 1**).

**Table 1:** Patient characteristics of cases reported in the literature and in our hospital.

Author/year/reference	Age (y)	Location	Mean diameter(cm)	Peripheral invasion	Shape	Uniformity of echo	Internal echo	Boundary	Color doppler flow imaging
Sherwin et al.1952 (6)	66	right	3.5	Yes	Oval	/	/	Ill-defined	/
Helm et al.1986 (7)	67	right	7	No	Lobulated	/	/	Well-defined	/
Wegner et al.1994 (8)	70	/	4.3	/	Oval	heterogeneous echo	hypoecho	/	/
Frates et al.1997 (9)	43	/	2.1	Yes	/	homogeneous echo	High-echo	Ill-defined	/
Varzaneh et al.2002 (10)	71	left	3.5	No	/	/	Mixed echo	Well-defined	/
Akbar et al.2003 (11)	/	left	2	Yes	Oval	heterogeneous echo	hypoecho	Ill-defined	/
Mechri et al.2009 (12)	78	left	6.5	No	/	/	hypoecho	Well-defined	/
Victor et al.2011 (13)	58	left	2.2	No	Lobulated	heterogeneous echo echo	hypoecho	/	abundant
Victor et al.2011 (13)	75	right	3.8	No	Lobulated	heterogeneous echo echo	hypoecho	Ill-defined	abundant
Muduly et al.2012 (14)	35	left	3	Yes	Lobulated	heterogeneous echo echo	High-echo	/	/
Krishnan et al.2020 (15)	60	right	4	Yes	Lobulated	/	hypoecho	Well-defined	abundant
Dehghani et al.2022 (16)	60	right	5	No	Lobulated	/	hypoecho	Well-defined	/
Wujin Hospital Affiliated to Jiangsu University.2022	58	left	3.5	No	Oval	heterogeneous echo	High-echo	Well-defined	abundant

"/":No relevant information.

According to the existing literature, epididymal leiomyosarcoma has common sonographic features of malignant tumors, such as solid changes, irregular shape, heterogeneous echo and with hypervascularity. Ultrasound imaging examination is helpful to differentiate benign epididymis lesions and can provide some reference for clinical diagnosis and treatment.

## 4 Discussion

The epididymis originates from the mesonephric duct of the embryo and is mainly composed of output tubules, epididymal ducts, and connective tissue. It is divided into three sections: head, body, and tail. The head and body regulate early and late sperm maturation, respectively, whilst the tail stores mature sperm. Epididymal leiomyosarcoma (EL) can develop at any age, with incidence peaking between 60 and 70 years (17). Leiomyosarcomas occur within organs containing smooth muscle, but are extremely rare in the male reproductive system. Patients usually present with a painless, hard mass, which may cause discomfort when invading nearby structures. On palpation, the mass is often well-circumscribed, lobulated, easy to move within the scrotum, and is sometimes accompanied by a small hydrocele (13).

The clinical manifestations of epididymal tumors are non-specific making it difficult for clinicians to distinguish between benign and malignant masses; therefore, patients are easily misdiagnosed. There is no effective method for the preoperative diagnosis of EL; diagnosis is dependent on postoperative pathology and immunohistochemistry. Macroscopically, the cut surface of a leiomyosarcoma appears gray, hard, and elastic. Microscopically, the cells contain bundles of polymorphic spindle cells, with heteromorphic nuclei, abnormal mitotic figures, and sometimes multinucleated giant cells. Histologically, tumor cells are diverse, ranging from well-differentiated to multi-type undifferentiated tumor cells. Immunohistochemistry is required to determine the presence of smooth muscle (18).

Ultrasonic examination is the most commonly used method in the diagnosis and differential diagnosis of reproductive system diseases due to its obvious advantages of convenience, real-time dynamic imaging and non-radiation. However, there is still controversy on the differential efficacy of benign and malignant epididymis space occupying lesions. Frates M C et al.’s research suggests that ultrasound is ineffective in differentiating benign from malignant epididymal tumors (9). Several studies have shown that ultrasound has important value in the localization diagnosis, internal echo, boundary and blood flow of paratesticular tumors. Paul S et al. reported that ultrasound can accurately locate the paratesticular lesions, and it is valuable to distinguish solid or cystic features (19). Derchi LE et al. reported that most solid scrotal tumors have no special features to help identify their nature. However, ultrasound can identify lesions in almost all cases, accurately estimate their scope and relationship with adjacent tissues, and diagnose some common benign scrotal lesions (20). The study of Mustafa S et al. shows that in most cases, malignant and benign paratesticular masses can be distinguished by ultrasound, MRI or combination, but it should also be noted that the imaging manifestations of benign and malignant tumors may overlap (21). Shaodong Qiu et al. proposed that ultrasound elastography is expected to become a new examination method for clinical diagnosis of scrotal diseases (22).

Beginning in 1949, when the first primary epididymal leiomyosarcoma was reported (23), we identified 41 relevant full text or abstract studies, with 11 (12 cases) featuring ultrasonic imaging characteristics. By analyzing the characteristics of ultrasound images in the existing literature, we have been able to draw conclusions on the appearance of epididymal leiomyosarcomas. These tumors are almost solid masses, with heterogeneous internal echogenicity and hypervascular. More than half of the tumors demonstrated irregular shape and unclear boundaries. Whilst local tissue invasion was common, no distant metastases were observed in any of the cases. In the literature we reviewed, although only 4 cases had information about the internal blood supply of the tumor, the result was with significant vascularity. Therefore, we speculate that EL has the characteristics of hypervascular. Ultrasound Doppler blood flow imaging technology and contrast-enhanced ultrasound technology are sensitive to blood flow display and can be used as a powerful tool to evaluate the distribution of tumor blood supply.

Given the different treatment methods required, it is important to differentiate epididymal leiomyosarcoma from benign epididymal tumors. Adenomatoid tumors are the most common benign mesothelial tumors and are often associated with no clinical symptoms (24). Their histological findings vary from those of epididymal leiomyosarcoma: a low nucleo-cytoplasmic ratio of cuboidal epithelial cells, cytoplasmic vacuolization, and small nucleoli. They may also exhibit fibrosis, hyalinization, and interstitial and lymphatic aggregation. The histopathological characteristics of adenomatoid tumors are reflected in the low, equal, high, and mixed echogenicity on ultrasonic images. Echoes are often heterogeneous, and ultrasonic manifestations lack specificity. However, the literature reports that the blood supply to adenomatoid tumors is not rich—a distinguishing feature on ultrasound imaging.

Leiomyomas are composed of single smooth muscle cells, arranged in bundles or braids. The clinical manifestations of epididymal leiomyoma and EL are similar; however, there are significant differences in diagnosis, treatment, and prognosis. Clear boundaries and multilayer spiral echoes are characteristic sonographic manifestations of leiomyomas; therefore, ultrasonography is valuable in the diagnosis of this tumor.

Smart et al. (25)summarized some common features of benign epididymal lesions: localized, painless, slow-growing. Ultrasound imaging of benign epididymal masses demonstrated regular shapes, clear boundaries, and uniform echoes, with minimal blood flow demonstrated by color Doppler flow imaging. As most EL are characterized by hypervascularity, Doppler imaging is a useful tool in differentiating these tumors from benign lesions.

Epididymitis is a common, nonspecific infectious diseases of the male reproductive system that may be misdiagnosed as EL. Acute epididymitis has typical clinical manifestations—scrotal swelling and pain that can radiate to the lower abdomen and thighs, poor cold tolerance, fever, and elevated white blood cell count. Ultrasound demonstrates enlarges testis and epididymis, with reduced internal echogenicity that appears heterogeneous. Color Doppler flow imaging shows abundant blood flow in the epididymitis, its clinical manifestations combined with ultrasonic imaging were easy to differentiate from EL. Ultrasound images of chronic epididymitis often show heterogeneous echogenicity and unclear boundaries, with reduced or no blood flow signals in some areas on Doppler imaging. This can lead to misdiagnosis and confusion with EL. Other clinical examination data, such as a history of acute epididymitis, should be integrated to avoid misdiagnosis.

Granulomatous inflammation is chronic proliferative inflammation characterized by granulomas, secondary to infection or autoimmune disease. The diversity of causes leads to varied and complex histology, morphology and sonographic findings, making diagnosis difficult. However, most granulomatous inflammations show hypovascularity on ultrasonography, allowing them to be distinguished from EL.

In recent years, the incidence rate of urinary tuberculosis has increased due to the increasing prevalence of drug-resistant tuberculosis. The epididymis is the most vulnerable part of the male reproductive system to tuberculosis infection; Drudi et al. (26) reported that epididymal tuberculosis mostly occurs in the epididymal tail. Ultrasonography of an epididymis with tuberculosis demonstrates heterogeneous echogenicity, with many small hypoechoic areas suggestive of granulomas. The combination of highly echogenic and anechoic areas suggests caseous necrosis and microcalcification. Calcification can be used as a specific manifestation to differentiate epididymal tuberculosis from other diseases, including EL. The blood supply of epididymal tuberculosis changes with the development of the disease, and the blood flow is rich during the proliferative period. When caseous necrosis, fibrous hyperplasia, and calcification completely destroy the internal structure of the epididymis, the blood flow is compromised. Granulomas and inflammation of the surrounding tissues result in annular blood flow signal. Contrast-enhanced Ultrasound(CEUS) shows circular enhancement, and some scholars believe that annular enhancement may be a characteristic manifestation of CEUS in epididymal tuberculosis.

In summary, Epididymal leiomyosarcoma is a rare disease with atypical clinical manifestations and difficult differential diagnosis., making it easy to confuse with benign and other malignant tumors. By reviewing the existing literature and analyzing the cases found in our institute, we summarized the ultrasonic characteristics of ELare solid changes, irregular shapes, heterogeneous internal echogenicity, and hypervascular. This is helpful to differentiate benign and malignant tumors of epididymis. Ultrasonography can provide some reference for clinical diagnosis and treatment. However, compared with other malignant tumors of the epididymis, it has no characteristic sonographic features.

## Data availability statement

The original contributions presented in the study are included in the article/supplementary material. Further inquiries can be directed to the corresponding authors.

## Ethics statement

Ethical review and approval was not required for the study on human participants in accordance with the local legislation and institutional requirements. Written informed consent for participation was not required for this study in accordance with the national legislation and the institutional requirements. Written informed consent was obtained from the individual(s) for the publication of any potentially identifiable images or data included in this article.

## Author contributions

RS, JX and HS performed the experiments, analyzed the data, and wrote the manuscript. ZX and HL were involved in performing the experiments. XY conceived the study and assumed overall responsibility for this work. All authors contributed to the article and approved the submitted version.
